# Rehabilitation Surgery for Peripheral Facial Nerve Injury after Facial Trauma

**DOI:** 10.1055/s-0044-1782199

**Published:** 2024-04-12

**Authors:** An Quang Lam, Thuy Phan Chung Tran, Duong Van Tran, Hiep Xuan Tran, Albert J. Fox, Luan Viet Tran

**Affiliations:** 1 Department of Otorhinolaryngology, Pham Ngoc Thach University of Medicine, Ho Chi Minh, Viet Nam; 2 Department of Aesthetic and Plastic Surgery, Cho Ray Hospital, Ho Chi Minh City, Ho Chi Minh, Viet Nam; 3 Department of Plastic and Microsurgery, Ho Chi Minh City National Hospital of Odontology, Ho Chi Minh City, Viet Nam; 4 Department of Facial Plastic Surgery, Southcoast Hospitals Group, New Bedford, Massachusetts, United States

**Keywords:** facial paralysis, nerve anastomosis, nerve grafting, sural nerve, facial trauma

## Abstract

**Introduction** Facial trauma can cause damage to the facial nerve, which can have negative effects on function, aesthetics, and quality of life if left untreated.

**Objective** To evaluate the effectiveness of peripheral facial nerve direct end-to-end anastomosis and/or nerve grafting surgery for patients with facial nerve injury after facial trauma.

**Methods** Fifty-nine patients with peripheral facial nerve paralysis after facial injuries underwent facial nerve rehabilitation surgery from November 2017 to December 2021 at Ho Chi Minh City National Hospital of Odontology.

**Results** All 59 cases of facial trauma with damage to the peripheral facial nerve underwent facial nerve reconstruction surgery within 8 weeks of the injury. Of these cases, 25/59 (42.3%) had end-to-end anastomosis, 22/59 (37.3%) had nerve grafting, and 12/59 (20.4%) had a combination of nerve grafting and end-to-end anastomosis. After surgery, the rates of moderate and good recovery were 78.4% and 11.8%, respectively. All facial paralysis measurements showed statistically significant improvement after surgery, including the Facial Nerve Grading Scale 2.0 (FNGS 2.0) score, the Facial Clinimetric Evaluation (FaCE) scale, and electroneurography. The rate of synkinesis after surgery was 34%. Patient follow-up postoperatively ranged from 6 to > 36 months; 51 out of 59 patients (86.4%) were followed-up for at least 12 months or longer.

**Conclusion** Nerve rehabilitation surgery including direct end-to-end anastomosis and nerve grafting is effective in cases of peripheral facial nerve injury following facial trauma. The surgery helps restore nerve conduction and improve facial paralysis.

## Introduction

In developing countries, trauma resulting from traffic accidents, occupational accidents, and violence is a major concern for society. In cases of hospitalization due to trauma, facial trauma accounts for 16% of cases. 1 Facial trauma can present a diverse range of injuries, including soft-tissue wounds, vascular injuries, bone fractures, and facial paralysis due to damage to the facial nerve.

Although accounting for only 6.3 to 6.83% 2 3 of traumatized hospitalization, patients with facial paralysis due to facial injuries should be diagnosed and treated early to avoid late sequelae. If not detected and treated promptly, damage to the facial nerve can result in serious functional, aesthetic, and quality-of-life impairments for the patient.

The injured facial nerve due to trauma must be restored with the aim of reestablishing nerve conduction in the damaged segment of the nerve, thereby re-establishing nerve stimulus to the facial muscles, improving facial symmetry at rest and during movement, and preventing complications caused by facial paralysis. There are various methods for reestablishing nerve conduction, including end-to-end anastomosis or nerve grafting, which are used for new nerve injuries.

## Methods

**Study design:** retrospective study

***Subjects and methods:****All* patients with peripheral facial paralysis after facial injuries underwent facial nerve rehabilitation surgery from November 2017 to December 2021

**Inclusion criteria:** All cases met the following criteria:

-Facial trauma
-Clinical and electroneurography (ENoG) evidence of facial paralysis
-Indication for facial nerve reconstruction surgery.

### Evaluation of Surgical Results

Patients were assessed for facial paralysis according to objective measurement using the Facial Nerve Grading Scale 2.0 (FNGS 2.0), self-assessment of quality of life using the the Facial Clinimetric Evaluation (FaCE) scale, 4 and ENoG before and at least 6 months after surgery.

For the FNGS 2.0 measurement, the total score was calculated, and the patients would be stratified from facial paralysis grade I to VI ( Table 1 ). For the FaCE scale, the total score was counted. For ENoG assessment, we use the ENoG value, which measures the degree of facial nerve degeneration on the paralyzed side and compares the proportion of the compound muscle action potential (CMAP) on a side with paralysis to the value on the normal face (counted in the form of percentage). 5

**Table 1:** Facial nerve grading scale 2.0 (FNGS 2.0) 1 6

Facial Nerve Grading Scale 2.0				
Score	Region			
	Brow	Eye	NLF	Oral
1	Normal	Normal	Normal	Normal
2	Slight weakness > 75% of normal	Slight weakness > 75% of normal Complete closure with mild effort	Slight weakness > 75% of normal	Slight weakness > 75% of normal
3	Obvious weakness > 50% of normal Resting symmetry	Obvious weakness > 50% of normal Complete closure with maximal effort	Obvious weakness > 50% of normal Resting symmetry	Obvious weakness > 50% of normal Resting symmetry
4	Asymmetry at rest < 50% of normal Cannot close completely	Asymmetry at rest < 50% of normal	Asymmetry at rest < 50% of normal	Asymmetry at rest < 50% of normal
5	Trace movement	Trace movement	Trace movement	Trace movement
6	No movement	No movement	No movement	No movement
Secondary movement (global assessment)			**Grade**	
0		None	**I**	**4**
1		Slight synkinesis; minimal contracture	**II**	**5–9**
2		Obvious synkinesis; mild to moderate contracture	**III**	**10–14**
3		Disfiguring synkinesis; severe contracture	**IV**	**15–19**
Reporting: sum scores for each region and secondary movement. NLF, nasolabial fold			**V**	**20–23**
			**VI**	**24**

The recovery degree of facial paralysis according to FNGS 2.0 was measured based on the difference of total score before and after surgery divided by the total score before surgery in form of %. The recovery degree is shown in Table 2 .

**Table 2:** The degree of facial paralysis recovery based on FNGS 2.0

%	Degree
0	No recovery
0< to <25	Poor recovery
25 to <50	Moderate recovery
≥50	Good recovery

### Surgical Methods

Operative indications for surgery include: 1) Traumatic wounds within zones 1 to 4 of the face; 7 2) Patients having FNGS 2.0 in at least one zone, greater than or equal to 4; 3) Patients with severe axonal damage on Electroneurography. Incision of the skin is made through an existing wound. Another preauricular incision is performed if necessary to expose the main trunk of the damaged nerve.

**Fig. 1:**
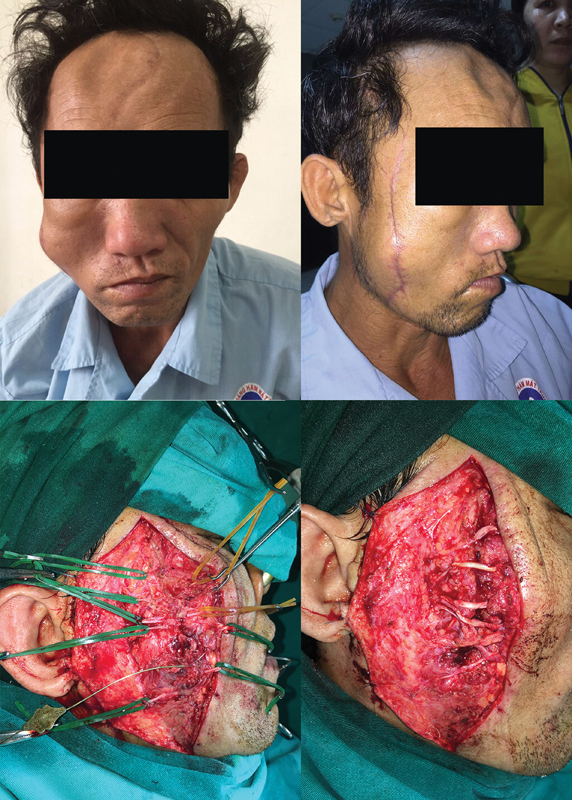
Exposure of the injured facial nerve branches on the right side. (1) right facial weakness; (2) existing scar; (3) affected branches, including one frontal branch, three zygomatic branches, and one buccal branch; (4) after nerve grafting for these five affected branches.

Based on the structure and anatomy of the branches of the peripheral facial nerve on the surface of the face, including the forehead, temporal area, cheeks, jaw, and other facial structures such as the prominence of the cheekbone, the angle of the mandibular bone, the parotid gland, and the parotid duct, it is possible to predict and connect the two ends of the damaged facial nerve branches together. 7 Once the terminal ends of the injured branches have been found, the damaged zone of the nerves should be evaluated to decide if any segments need to be removed. If the nerve ends are cut sharply and appear healthy, there may be no need to refresh them. However, if the nerve ends are compressed or damaged, a scalpel is used to cut the nerve to the point where it is healthy. At this point, the nerve fibers will protrude slightly beyond the external sheath of the nerve (the epineurium). After removing the damaged part of the nerve, if the distance between the two ends of the injured nerve is too long, it is necessary to dissect and free up more length of the nerve on both sides. Sometimes, subcutaneous sutures may be needed to bring the two ends of the nerve closer together. The extent of the deficit can then be determined by measuring the distance between the two ends of the nerves. Fig. 1 demonstrates the exposure of the injured nerves and repair with nerve grafting.

### Direct End-to-end Anastomosis

When the gap between the two damaged nerve ends is less than 14 mm, the surgeon should try to bring them together and evaluate the tension. If the tension is not excessive, the two ends of the injured facial nerve can be directly sutured together.

The nerve repair is performed under surgical loupes with a magnification of 4.5-to-6 times. We use epineural repair for direct end- to- end anastomosis. The suturing is performed on the external sheath of the nerve, starting from one end, and progressing to the other. After the first knot is tied, the ending suture of the knot is held to keep a slight tension on the anastomosis, allowing for taking and tying the second knot next to it. The suturing continues until the two ends of the nerve are completely connected.

The sutures used for nerve repair are typically 8–0 nylon for the main and common trunks, or 9–0 for the branches or sub-branches of the facial nerve, using the technique of epineural repair suture.

### Nerve Graft Repair

When the extent of the deficit of the branch of the facial nerve is longer than 14 mm, a nerve graft repair is performed. The total number of damaged nerve branches and the total length of the missing segments should be determined to select a suitable nerve graft donor.

**Fig. 2:**
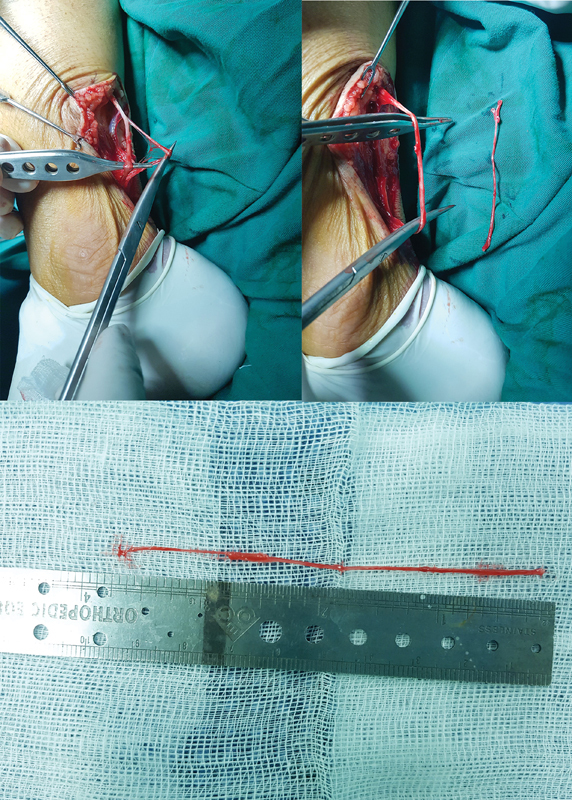
Harvesting of the sural nerve. (1) the dissect the nerve from its accompanying vein; (2) dissect a portion of sural nerve; (3) nerve graft.

The sural nerve is chosen as nerve graft. The ankle joint is bent at a 90-degree angle and tilted toward the opposite thigh. The skin behind the lateral malleolus is incised, ∼ 1 to 2 cm from the Achilles tendon. A segment of skin measuring 2 to 8 cm in length should be incised, depending on the number of injured nerves that require grafting. The sural nerve and a small accompanying vein are exposed under the skin. It is necessary to dissect the sural nerve from the vein and adjacent tissues. The partial sural nerve is harvested, with the extent of the harvested nerve dependent on the need for nerve grafting. After identifying the portion of the sural nerve with a diameter comparable to the damaged segment of the facial nerve, the nerve can be further dissected superiorly and inferiorly along its trunk to achieve the desired length. Fig. 2 demonstrates the process of harvesting the sural nerve.

**Fig. 3:**
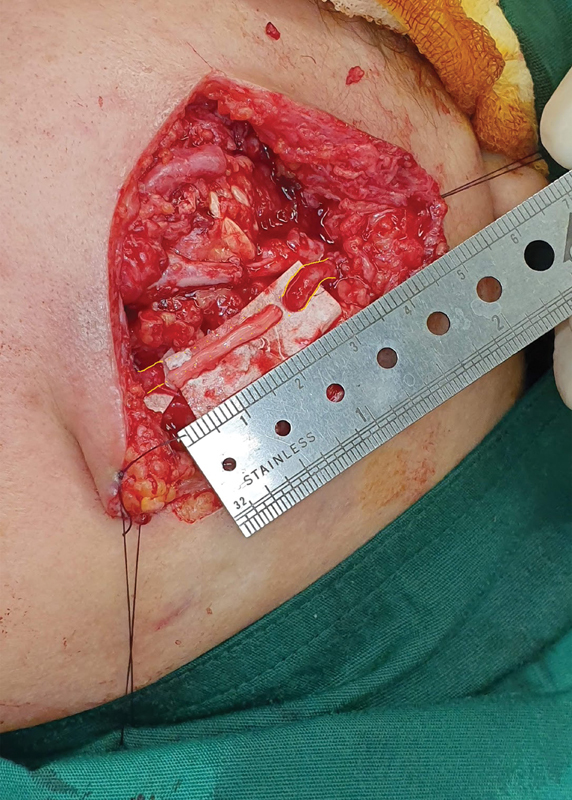
The harvested nerve graft is placed between the two ends of the damaged nerve to make sure that the nerve segment after joining is not stretched. (The green part is the sural nerve graft, and the yellow part is the injured buccal branch).

After the nerve graft is harvested, the two ends of the graft should be cut using a scalpel or scissors and placed on a wet gauze pad to prepare for nerve repair. The two ends of the graft should then be placed between the two severed ends of the damaged facial nerve, ensuring that the repaired nerve is not under tension (see Fig. 3 ). The nerve ends should be sutured together using the epineural suture, until the two ends of the nerve are completely anastomosed.

## Result

Between November 2017 and December 2021, there were 59 cases of facial trauma with facial nerve paralysis. The average age of the patients was 32.4 ± 10.6 years, with the youngest patient being 14 years old and the oldest being 58 years old. Most cases were male, accounting for 48 out of 59 cases (81%). The main causes of facial trauma were physical assault in 26 cases (44.1%), followed by traffic accidents in 19 cases (32.2%) and occupational accidents in 14 cases (23.7%). Of these injuries, 32 cases (54.2%) were penetrating trauma, 24 cases (40.7%) were blunt trauma, and 3 cases (5.1%) were combined. All patients underwent pre-operative CT scan or 3D CT scan to evaluate for possible facial fractures. Concurrent facial fractures were identified in 21 cases (37.3%). Disruption of the parotid duct was found in 40 cases (67.8%). The average time from injury to surgery was 18.3 ± 9.8 days, ranging from 18 hours to 44 days, and most patients undergo surgery after a week (97%). The average follow-up period was 31.8 ± 16.3 months, of which the shortest follow-up was 6.4 months and the longest was 57.67 months; 51 of 59 patients (86.4%) were followed up for at least 12 months or longer and 8 patients (13.6%) were followed up for 6 to 12 months.

### Surgical Methods

Twenty-five out of 59 patients underwent direct end-to-end anastomosis while 22/59 cases received nerve graft repair only. And we combined both direct end-to-end anastomosis and nerve graft for 12 cases (see Table 3 for summary of surgical methods).

**Table 3:** Surgical methods

Surgical methods	Number of cases *N* = 59 (100%)
Direct end-to-end nerve anastomosis	25/59 (43%)
Nerve graft	22/59 (37%%)
Combined method	12/59 (20%)

There was a total of 150 facial branches affected in 59 patients, including 2 main trunks, 21 frontal branches, 50 zygomatic branches, 71 buccal branches, and 6 marginal branches. In which, 81 branches had direct end-to-end anastomosis, while 69 branches had nerve grafts. Table 4 outlines the facial nerve branches affected, and type of surgical repair performed (direct end-to-end repair or nerve graft).

**Table 4:** Total number of damaged facial branches and surgical repair method

Facial nerve branches	Direct end-to-end ( *n* = 81)	Nerve graft ( *n* = 69)	Total
Main trunk	1	1	2
Frontal branches	9	12	21
Zygomatic branches	24	26	50
Buccal branches	41	30	71
Marginal branches	6	0	6

### Improvement of Facial Paralysis after Surgery

All the facial paralysis assessment scores improved significantly after surgery including FNGS 2.0, FaCE scale, and ENoG ( Table 5 ).

**Table 5:** Comparison between before and post-op facial nerve repair surgery

Features	Before		After surgery		*P* *
	Mean ± CI	Median (quartile)	Mean ± CI	Median (quartile)	
**FNGS 2.0**	16.8 ± 4.4	14 (14–21)	7.2 ± 3.2	7 (4–9)	< 0.001
**FaCE**	0.4 ± 0.2	0.43 (0.3–0.5)	0.8 ± 0.1	0.9 (0.8–1)	< 0.001
**ENoG**	0.1 ± 0.1	0 (0–0.1)	0.6 ± 0.3	0.6 (0.3–0.9)	< 0.001

Abbreviations: CI, confidence interval; ENoG, electroneurography; FaCE, Facial Clinimetric Evaluation; FNGS 2.0, Facial Nerve Grading Scale 2.0.

* Wilcoxon Sign-Rank test.

**Fig. 4:**
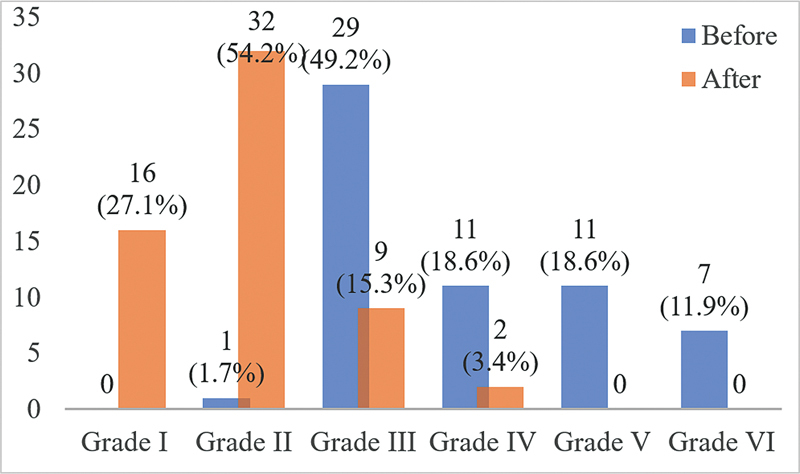
Degree of facial paralysis before and after surgery according to FNGS 2.0.

Before surgery, most patients had severe facial paralysis, from grade III to VI. After surgery, there was a significant improvement in facial paralysis. Facial nerve function became normal in 16 patients (27.1%) after surgery ( Fig. 4 ).

**Fig. 5:**
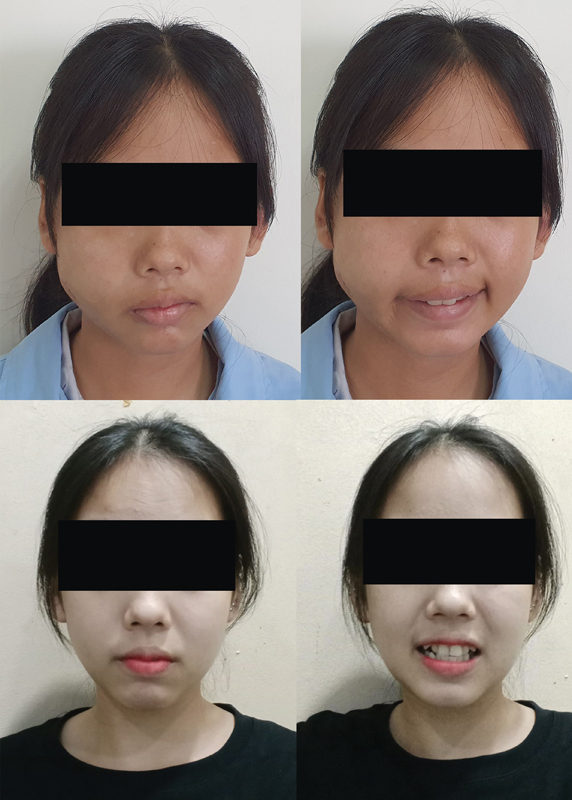
Patient after surgery improved from grade III paralysis (top) to grade I (bottom).

Fig. 5 demonstrates a patient's improvement after surgery from grade III paralysis to grade I.

**Fig. 6:**
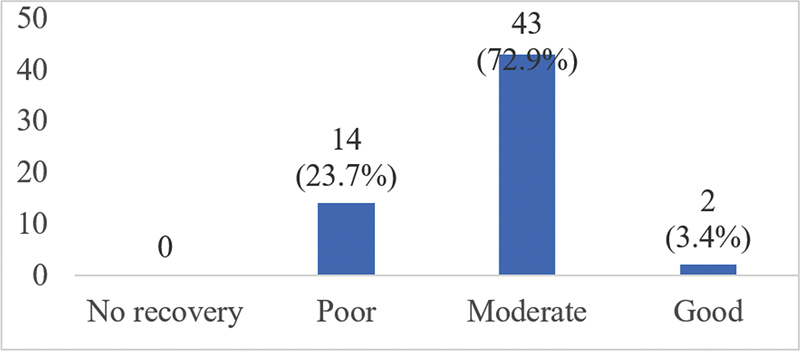
Degree of recovery after facial nerve repair surgery based on FNGS 2.0.

All patients had facial nerve recovery postsurgery. Most cases, 43/59 patients (72.9%) had moderate recovery based on FNGS 2.0 score ( Fig. 6 ).

The end-to-end anastomosis group had the best improvement in facial paralysis after surgery (according to FNGS 2.0, FaCE scale and ENoG), followed by the combined group and the nerve graft group had the least. This difference was statistically significant ( Table 6 ).

**Table 6:** Surgical methods and their outcome

	Surgical methods			*P* *
	End-to-end ( *n* = 25)	Nerve graft ( *n* = 22)	Combined ( *n* = 12)	
**FNGS 2.0**	6 ± 2.9	8 ± 3	7.8 ± 3	0.015
**FaCE scale**	0.9 ± 0.11	0.82 ± 0.13	0.89 ± 0.1	0.019
**ENoG**	0.71 ± 0.25	0.47 ± 0.22	0.65 ± 0.25	0.004

Abbreviations: ENoG, electroneurography; FaCE, Facial Clinimetric Evaluation; FNGS 2.0, Facial Nerve Grading Scale 2.0.

* : Kruskal Wallis test.

### Complications

No major complications were noted after surgery. Synkinesis occurred in 20 cases (33.9%), in which 12 cases (20.3%) were minor and 10 cases (16.9%) were moderate.

## Discussion

### Time of Surgery

The likelihood of nerve injury is high with sharp wound injuries because branches of facial nerves lie superficially under the skin, and it is necessary to expose the nerve for repair. The timing of repair may vary and be influenced by many factors, including the patient's health status, associated injuries, the extent of damage, and the availability of surgical resources and surgeon's experience. In contrast, for blunt injury and partial paralysis, internal medicine and physical therapy can be used for treatment. 8 9

The timing of nerve repair is divided into acute repair (within 72 hours after injury), subacute repair (after 72 hours to 6 months), and delayed repair (after 6 months of injury). 10 Within the first 72 hours, the distal end of the motor fibers can still be stimulated, which helps to detect and repair the nerve accurately. Furthermore, in nerve injuries less than 72 hours, the nerve has not yet atrophied, and the surrounding tissue has not yet scarred, so it is easier to find the injured nerve during surgery. After 72 hours, due to the atrophy of the nerve distal to the injury site, the muscle at the site of injury will no longer be stimulated. At this point, nerve repair needs to be based on knowledge of nerve anatomy at the injury site. 11 The later the surgery, the more difficult it is to find the injured nerve due to the formation of scar tissue around the damaged nerve ends.

In our study, the average time from injury to surgery was 18.3 ± 9.8 days, with most cases undergoing surgery between 72 hours and 1.5 months after injury (57/59), and only 2 cases undergoing surgery on the first day (2/59).

Susan and Fliss agree that early management of nerve injuries within 72 hours is important for restoring nerve conduction. 12 13 Additionally, McElwee also suggests that efforts should be made to surgically restore nerve conduction, even for delayed nerve injuries up to 12 months, as muscle function can still be restored. 14 After 12 months, the muscle is atrophied, and even surgical nerve repair will not improve muscle function.

In a recent recommendation during the coronavirus disease 2019 (COVID-19) pandemic, Faramarzi suggests that there is no straightforward evidence that nerve repair surgery for the facial nerve before 72 hours will result in better recovery than surgery performed between 72 hours and 2 weeks. 15 However, if the patient is currently infected with severe acute respiratory syndrome coronavirus 2 (SARS-CoV-2), the risk of mechanical ventilation or death after surgery will be higher. In this case, waiting for 72 hours to screen for COVID infection may result in better overall surgical outcomes.

### Surgical Methods

In our study group, 25 cases (42.3%) underwent end-to-end anastomosis, 22 cases (37.3%) underwent nerve graft techniques, and 12 cases (20.4%) underwent combined end-to-end anastomosis and nerve grafting. Most cases underwent surgery more than 72 hours after injury, at which point the nerve endings had undergone some degree of atrophy, and the scar tissue at the injury site caused the two ends of the nerve branch to be distant from each other. Locating the two damaged nerve endings during surgery was also challenging because the nerve was fragmented and had a similar appearance to the scar tissue around it. Based on the surface anatomical landmarks of the facial nerve in relation to certain fixed facial structures which has been described in our previous study, two damaged nerve endings are identified and matched successfully in all cases. 7 Once the two damaged nerve endings were found, a relatively wide damaged zone of the nerve needed to be removed, making end-to-end anastomosis more difficult than in cases of fresh nerve injury.

Therefore, we emphasize that the timing of surgery plays an important role in deciding the surgical methods for cases of facial nerve injury due to trauma, in agreement with the findings of Brown et al. and Volk et al. 16 17 Volk documented that cases of nerve damage later than 2 months are difficult to perform anastomosis or nerve grafting as the distal end of the damaged nerves may not be visible. Consequently, the author proposed performing hypoglossal-facial nerve or cross-facial nerve transplantation instead. 16 In our study, all cases were performed within 2 months, and both ends of the damaged nerve branches were found.

The most common donor nerve graft reported by Li et al. was the great auricular nerve 18 for repairing the facial nerve after tumor resection involving the facial nerve as its convenience without making an additional skin incision. However, in our practice, we prefer to use the sural nerve because of its similar diameter to the facial nerve branches (between 2.3–2.8 mm) and the minor complications associated with harvesting a single segment of the sural nerve. Additionally, the sural nerve graft can be harvested up to 40 cm in length, making it an excellent source for nerve grafting, as noted in previous studies. 19 20 21

Other facial injuries encountered included parotid duct injury and facial fractures. Parotid ductal injury and fractures were managed and repaired at the same time as the facial nerve repair. Disruption of the parotid duct occurred in 40 cases (67.8%). In cases in which parotid duct injury was uncertain, sialography was used to confirm duct integrity. Repair was performed after inserting a small canula through the Stenson duct to aid in repair and in determining the course of the duct. Facial bone fractures were found in 21 cases (37.3%), and there were no temporal bone fractures.

### The Outcome of Rehabilitation Surgery

Min Hu et al. (2016), Kannan et al. (2020), Fabienne Carré et al. (2022), and Fliss et al. (2022) have performed nerve restoration surgery for patients with facial paralysis caused by injuries, tumor resection, or other pathological conditions and surgical accidents, with surgical timing ranging from acute to subacute and up to 5 years after the injury. 12 22 23 24 These authors mostly used nerve grafting techniques to achieve mild-to-moderate facial paralysis after surgery ( Table 7 ).

**Table 7:** Relationship between wound features, surgical methods, and the improvement of facial paralysis

	N	Time of surgery	Wound features	Surgical methods	Improvement of facial paralysis
Min Hu (2016) 2	7	6–21 days	Facial injuries, after surgery	Sural nerve graft	HB II: 71.4%; HB III: 28.6%
Kanna (2020) 3	18	Early (before 48 hours): 8 cases; late (7 months–5 years): 10 cases	After surgery and injuries	Direct end-to-end anastomosis, vascular nerve grafting, nerve transfer	Good improvement: 61.1%. No improvement: 16.7%
Fabienne Carré (2022) 4	31	Before 48 hours: 3 cases; Late: 29 cases	Injuries, after surgery, tumors, pathologies	Direct end-to-end anastomosis: 2 cases, nerve graft: 29 cases	HB III: 58.1%; HB IV: 6.5%; HB VI: 35.4
Fliss (2022) 5	12	Acute: 9 cases; subacute (< 24 weeks)	Injuries, postoperative, pathological	Direct end-to-end anastomosis: 1 case, nerve graft:11 cases	58.3% return normal
Our study	59	Acute: 2 cases; subacute (< 1.5 months): 57 cases	Facial injuries	Direct end-to-end anastomosis: 25 cases; nerve graft: 22; combined: 12 cases	HB I–II: 27: 81.3%; HB III: 15.3%; HB IV: 3.4%

In our study, all patients suffered from facial trauma and underwent surgery within 45 days after the injury. The results of facial nerve restoration surgery depend on several factors, with the most important being the functional status of the distal end of the injured nerves, the non-atrophied state of facial muscle, and the unaffected nerve conduction. 25 In theory, nerve conduction redistribution should be restored early, ideally within the first 72 hours after injury, as the muscle end plate and facial muscles are still Intact. All patients also received pre and postoperative injectable steroids. Steroids were used to reduce inflammation and edema of the facial nerve which may impact facial nerve function and outcome.

In our study, the direct end-to-end anastomosis group had 25 cases with the lowest average FNGS 2.0 score of 6 ± 2.9, followed by the combined end-to-end anastomosis, and the nerve graft group. The group with nerve graft had the highest average FNGS 2.0 score. This difference is statistically significant and suggests that direct end-to-end anastomosis, if possible, can bring the most significant improvement after surgery. Similarly, Kannan noted that there were cases with nerve grafting surgery performed within the first 24 hours after the injury but no improvement in facial paralysis was seen after surgery, while cases with delayed end-to-end anastomosis surgery still achieved complete restoration. 23

Timing of surgery is an essential factor in selecting the proper surgical method. Patients who undergo surgery earlier after injury have less scar tissue and the injured nerves are less degenerated. This makes it easier to find the affected nerves during surgery and direct end-to-end anastomosis can be performed easily without the need for nerve graft. Consequently, the recovery of facial paralysis after surgery is better.

## Conclusion

Nerve rehabilitation surgery, including direct end-to-end anastomosis and nerve grafting, is effective in cases of peripheral facial nerve injury following facial trauma. The surgery helps restore nerve conduction and improve facial paralysis and limit the development of postparalysis sequelae.
